# Expression of Macrophage Polarization Markers against the Most Prevalent Serotypes of *Aggregatibacter actinomycetemomitans*

**DOI:** 10.3390/microorganisms10071384

**Published:** 2022-07-09

**Authors:** Daniel Betancur, Camila Muñoz, Angel Oñate

**Affiliations:** 1 Laboratory of Molecular Immunology, Department of Microbiology, Faculty of Biological Sciences, Universidad de Concepción, Concepción 4030000, Chile; dbetancur@udec.cl (D.B.); camilapmunoz@udec.cl (C.M.); 2 Discipline of Periodontology, Department of Surgical Stomatology, Faculty of Dentistry, Universidad de Concepción, Concepción 4030000, Chile

**Keywords:** inflammation, periodontal cells, periodontal pathogens, periodontitis, innate immunity, intracellular bacteria

## Abstract

*Aggregatibacter actinomycetemcomitans*, a Gram-negative bacterium with seven serotypes (a–g) according to the structure of its LPS, has been defined as one of the most important pathogens in the development of a dysbiotic periodontal biofilm and the onset of periodontitis (an inflammatory chronic disease of the tissues around the teeth), where the serotype b is characterized as the most virulent compared with the other serotypes. The aim of this study was to evaluate the expression of the macrophage polarization markers M0, M1, and M2 against *A. actinomycetemcomitans*. Methods: THP-1 cells were infected with *A. actinomycetemcomitans* serotypes a, b, and c. The expression of CD11b, CD4, CD14, and CD68 for M0; IL-6, HLA/DRA, and CXCL10 for M21, and IL-10, CD163, fibronectin-1 or FN1, and CCL17 was evaluated by qPCR at 2 and 24 h after infection. Results: An increase in the expression of these molecules was induced by all serotypes at both times of infection, showing higher levels of expression to the M1 panel at 2 and 24 h compared to other markers. Conclusions: *A. actinomycetemcomitans* has a role in the macrophage polarization to the M1 phenotype in a non-serotype-dependent manner.

## 1. Introduction

The oral microbiome has been defined as a community of microorganisms of at least 1000 microbial species including bacteria, viruses, fungi, archaea, and protozoa that live in the oral cavity distributed according to the characteristics of each ecological niche available in the mouth [1,2]. This microbiome has been proposed as an etiological agent of various pathologies of the oral cavity such as cavities, gingivitis, periodontitis, and diseases associated with the placement of implants and intraoral medical devices [3,4]. However, the evidence available on bacterial populations at the oral level shows that the mere presence of bacteria is not enough to produce these pathologies [5], and that a disruption of the balance of the interactions between microbial communities and the host is necessary to produce the different pathologies [6].

In this sense, the term “*oralome*” has been coined to define all of these interactions that take place between the oral microbiome and the host during processes such as the maturation and development of an appropriate oral immune response, making evident the importance of the interaction between the host and pathogens in the initiation and development of highly prevalent diseases such as tooth decay or periodontitis, where, in many cases, the mechanisms of the disease are not only exclusive to the bacteria [7,8,9].

Within this type of pathology, periodontitis is currently defined as a non-communicable chronic, multifactorial, and inflammatory disease associated with a dysbiotic biofilm, with inflammation and destruction of the tooth-supporting and protection tissues, and with clinical attachment loss, alveolar bone destruction, the formation of periodontal pocketing, and gingival bleeding [10].

Periodontitis has an infectious etiology, associated with the development of pathogenic bacterial communities in the tissues surrounding the tooth, where approximately 700 cultivable oral bacterial species have been described, with 400 making up this subgingival biofilm, located in the virtual space between the tooth and the gum [11,12].

Many pathogens, such as *Porphyromonas gingivalis* and *Aggregatibacter actinomycetemcomitans*, defined as “*keystone pathogens*” due to their protagonist role in the development of the dysbiotic biofilm, have been linked with the progression and severity of this disease, the level of tissue destruction, and the subversion of the host’s immune system [13,14,15].

*A. actinomycetemcomitans* is a Gram-negative, non-motile, facultative anaerobic, and capnophilic coccobacillus present in both health and periodontal pathologies with a heterogeneous virulent behavior depending on the conditions available in the environment in which it is found [16,17]. The structural differences in its lipopolysaccharide (LPS) have led to the description, based on the composition of the O-polysaccharide chain of this structure including seven different serotypes (a–g) with serotypes a, b, and c the most prevalent in the oral cavity in humans [18,19].

In many studies, serotype b has been described to the most virulent and prevalent in periodontitis patients, and in different cell models it has been shown to induce a greater response in terms of the production of chemokines, cytokines, cytokine receptors, and tissue destruction molecules, such as metalloproteinases (MMP) or receptor activator nuclear kappa ligand (RANKL), by immune cells compared to the other serotypes present in the oral cavity [20,21,22,23,24]. Despite this, to this day there is little evidence about this differential character of the response produced by these serotypes in other cell lines such as stromal cells, e.g., keratinocytes or macrophages, or the effect of this variable virulence on phenomena such as the polarization to the M1 or M2 macrophage phenotypes during the progression and resolution stages of periodontitis.

In relation to this, extensive evidence has shown the ability of macrophages and other immune and structural cells to recognize periodontal pathogens, including *A. actinomycetemcomitans*, and produce cytokines, chemokines, antibacterial proteins, alarmins, and other molecules in response to this bacterium, thus being the first line of defense of the host at the level of the periodontal sulcus or pocket, where these cells are capable of moving transepithelial from the underlying connective tissue to the periodontal sulcus/pocket, taking direct contact with the microorganisms present there, and being of vital importance in the early stages of the settlement and colonization by microorganisms [25,26,27,28].

Nevertheless, the possible differential response of these cell types against the bacterial serotypes a, b, and c is not entirely clear yet, with little knowledge about whether this variable response is a characteristic only of the professional immune cells such as dendritic cells, lymphocytes, and others [20,21,22,23,24], or if the structural differences of this microorganism could have an effect at the level of the innate host defense from the earliest stages of infection, modulating in a serotype-dependent manner phenomena such as macrophage polarization, currently defined as key in the development and resolution of infectious and inflammatory processes.

This study aimed to analyze the expression of macrophage polarization markers (M0, M1, and M2) against *A. actinomycetemcomitans*, and to measure if this genic expression has a serotype-dependent differential character as described for other cell models [20,21,22]. For this, an in vitro infection model previously used by our team [29] was employed, using the most prevalent *A. actinomycetemcomitans* serotypes (a, b, and c) and macrophage cells to simulate the initial stage of periodontitis.

## 2. Materials and Methods

### 2.1. Bacteria Stains

The *A. actinomycetemcomitans* serotype a (ATCC^®^ 43717), serotype b (ATCC^®^ 43718), and serotype c (ATCC^®^ 43719) were incubated at 37 °C in capnophilic conditions (8% O_2_ and 12% CO_2_) in BHI broth (70138 GranuCult, Merck^®^, Darmstadt, Germany) added with 10% horse serum (H1270 Sigma-Aldrich^®^, Gillingham, UK) for 24 h based on the growth curves previously obtained by our group under standard conditions. For each experiment, bacteria were used at the exponential growth phase to obtain a consistent number of microorganisms with full immunogenic potential.

### 2.2. THP-1-Derived Macrophages Culture

The cell line ATCC^®^TIB-202 of human monocytic leukemia (THP-1) was cultured in RPMI-1640 medium (R8758 Gibco^®^, Carlsbad, CA, USA) supplemented with 10% fetal bovine serum (F2442 Sigma-Aldrich^®^) and pyruvic acid (107360 Sigma-Aldrich^®^, Gillingham, UK) (1 mM final concentration), glucose (G8270 Sigma-Aldrich^®^, Gillingham, UK) (14 mM final concentration), 2-mercaptoethanol (M6250 Sigma-Aldrich^®^, Gillingham, UK) (0.5 mM final concentration), 4-(2-hydroxyethyl)-1-piperazineethanesulfonic acid (HEPES) (H3375 Sigma-Aldrich^®^, Gillingham, UK) (10 mM final concentration. pH 7.35) and penicillin/streptomycin (15140-122 Gibco^®^, Carlsbad, CA, USA) (100 U/mL final concentration of penicillin and 100 μg/mL of streptomycin) using a humidified atmosphere at 37 °C and 5% CO_2_.

To produce differentiation into macrophagic cells, THP-1 cells were grown at the aforementioned conditions, according with the protocol previously described [30,31], using phorbol 12-myristate 13-acetate (PMA) (16561-29-8 Sigma-Aldrich^®^, Gillingham, UK) (100 nM final concentration) for 2 days.

### 2.3. Infection Assay

THP-1 cells were seeded using 1 × 10^6^ cells per well using 6-well plates and activated for 48 h as mentioned above. Bacteria were washed with phosphate-buffered saline solution (PBS), suspended in PBS, and added to THP-1 cells plates using approximately 100 bacteria as multiplicity of infection (MOI). The plates, to achieve the contact between the cells and the bacteria, were centrifuged for 10 min at 300× *g* (RCF). Plates were incubated for 90 min at 37 °C in 5% CO_2_ allowing for the internalization of bacteria in the macrophagic cells. Finally, cells were washed and incubated with fresh medium supplemented with metronidazole and gentamicin (G1914 Sigma-Aldrich^®^, Gillingham, UK) (M3761 Sigma-Aldrich^®^, Gillingham, UK), (200 μg/mL and 300 μg/mL, respectively) for 2 and 24 h defined as the post infection times.

### 2.4. RNA Extraction and RT-PCR

Total RNA was obtained from the THP-1 cells using TRIzol^®^ reagent (T9424 Invitrogen^®^ Sigma-Aldrich^®^, Gillingham, UK) according to the manufacturer’s protocol; 2000 ng of the extracted RNA were used in the reverse transcription reaction using the First-Strand cDNA Synthesis SuperMix kit (18080400 Invitrogen^®^. Thermofisher^®^, Waltham, MA, USA) following the manufacturer’s instructions for reverse transcription with DNase digestion step.

### 2.5. qPCR

Three panels of markers were used to define the macrophagic phenotypes. For M0 macrophages we used the expression of CD11b, CD4, CD14, and CD68. M1 were evaluated by the expression of interleukin 6 (IL-6), HLA class II histocompatibility antigen DR alpha chain (HLA/DRA), and C-X-C Motif Chemokine Ligand 10 (CXCL10), and, finally, M2 phenotype using the markers CD206, interleukin 10 (IL-10), CD163, fibronectin 1 (FN1), and C-C motif chemokine ligand 17 (CCL17).

The mRNA expression of the markers was measured using quantitative real-time polymerase chain reaction (qPCR). For this purpose, 30 ng of cDNA were amplified with the primers previously designed in the Primer-BLAST (NCBH-NIH) platform and Ensembl Genome, using the sequences shown in Table 1.

Takyon^®^ No Rox SYBR^®^ MasterMix dTTP Blue (UF-NSMT-B0701 Eurogentec^®^, Seraing, Belgium) reagent was used in an AriaMx Real-time PCR System (Agilent^®^, Santa Clara, CA, USA) using this thermal profile: 95 °C for 3 min, followed by 40 cycles of 90 °C for 5 s and 60 °C for 30 s, ending with a melt curve of 95 °C for 15 s, 60 °C for 1 min, and 95 °C for 15 s, for detection of non-specific amplification products that could lead to false positive signals. 18S rRNA expression levels were used as a normalizing endogenous control.

### 2.6. Statistical Analysis

The relative quantification was performed normalizing each gene’s mRNA expression to 18S rRNA expression by the 2^−∆∆Ct^ method [32]. The qPCR data were analyzed using the software GraphPad Prism 8.0 (GraphPad Software. Inc., San Diego, CA, USA). The normality distribution was measured using Kolmogorov–Smirnov test and the differences among groups were evaluated using Tukey’s test and two-way ANOVA analysis. Asterisks indicate a *p*-value considered statistically significant (*p* < 0.05). The data were expressed as fold-change means and standard deviation, from 3 independent experiments performed at different times as biological replicates, and qPCR reactions for each gene and sample were performed in duplicate as technical replicates.

## 3. Results

### 3.1. Expression of M0 Markers in Response to Aggregatibacter actinomycetemcomitans Serotypes

**Figure 1:**
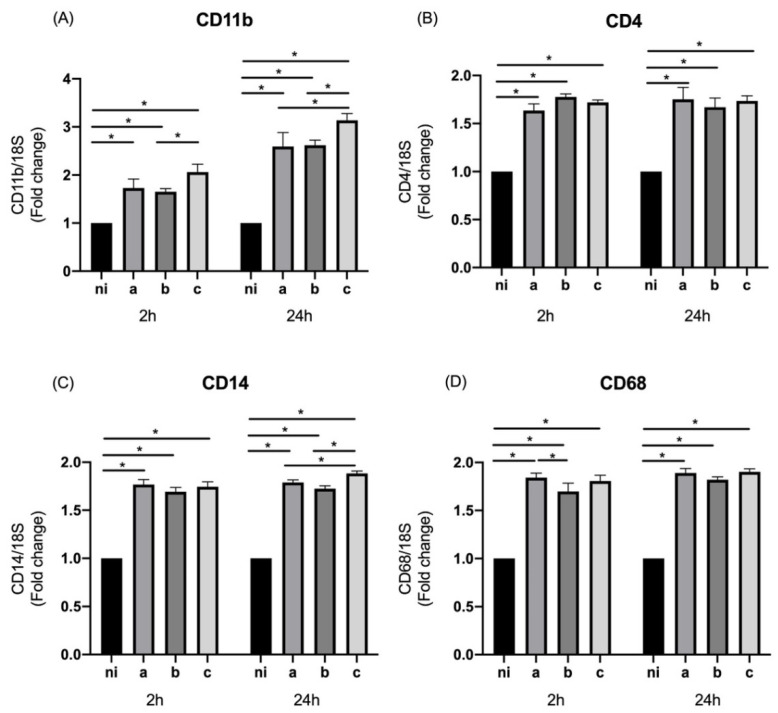
M0 markers’ expression by *Aggregatibacter actinomycetemcomitans*-induced THP-1 human macrophages (ATCC^®^ TIB-202TM). Expression in macrophages infected at an MOI = 102 with strains ATCC^®^ 43717TM (serotype a), ATCC^®^ 43718TM (serotype b), and ATCC^®^ 43719TM (serotype c), 2 and 24 h after infection. For relative expression, mRNA expression in non-infected (ni) macrophages was considered as 1, as a reference for fold-change in expression using 18S rRNA expression levels as a normalizing endogenous control. Data are represented as fold-change means and standard deviation of three independent experiments performed in duplicate. Asterisks were used to indicate a value of *p* considered statistically significant (* *p* < 0.05).

We evaluated the effect of *A. actinomycetemcomitans* serotypes on the expression of M0 markers by THP-1 cells. For the case of CD11b, a statistically significant overexpression was observed for the three serotypes evaluated compared to the non-infected control at 2 h (*p* = 0.0001, *p* = 0.0004, and *p* < 0.0001 for the serotypes a, b, and c, respectively) as well as at 24 h, when the three serotypes were compared with the non-infected condition (*p* < 0.0001 for all serotypes). Regarding the differences among the serotypes at 2 h, statistically significant differences were observed between serotypes b and c, with a higher expression induced by serotype b (*p* = 0.0194). On the other hand, after 24 h of infection there was a statistically significant greater expression for serotype c compared with serotype a (*p* = 0.0021) and b (*p* = 0.0033), without differences between the last two mentioned (*p* = 0.9962) (Figure 1A).

When the expression of CD4 was measured, a statistically significant higher expression was observed for the three serotypes studied at both times of infection compared with the control condition (*p* < 0.0001); however, no statistically significant differences were observed among the serotypes at either time of infection (Figure 1B).

In the case of CD14, statistically significant differences were observed for the three serotypes studied at both times of bacteria stimulation compared with the control condition (*p* < 0.0001). In terms of the differences among serotypes just at 24 h of infection, an overexpression of this molecule was induced by serotype c with respect to serotypes a (*p* = 0.0232) and b (*p* = 0.0002) (Figure 1C).

CD68 was measured and a statistically significant higher expression was observed for the three bacteria studied at 2 and 24 h of infection compared with the non-infected condition (*p* < 0.0001); however, statistically significant differences were observed among the serotypes only at 2 h of infection by serotype a over serotype b (*p* = 0.0087) (Figure 1D).

### 3.2. Expression of M1 Markers in Response to Aggregatibacter actinomycetemcomitans Serotypes

**Figure 2:**
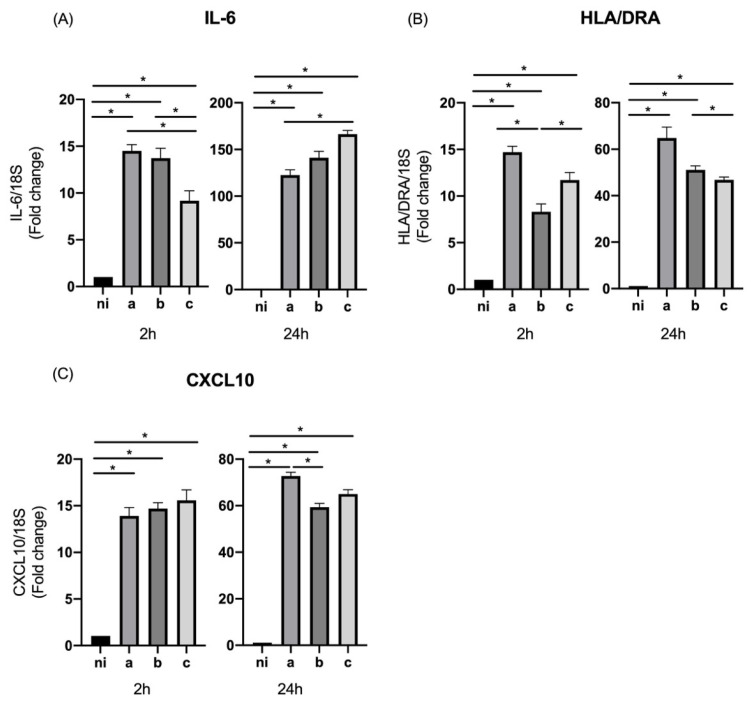
M1 markers’ expression by *Aggregatibacter actinomycetemcomitans*-induced THP-1 human macrophages (ATCC^®^ TIB-202TM). Expression in macrophages infected at an MOI = 102 with strains ATCC^®^ 43717TM (serotype a), ATCC^®^ 43718TM (serotype b), and ATCC^®^ 43719TM (serotype c), 2 and 24 h after infection. For relative expression, mRNA expression in non-infected (ni) macrophages was considered as 1, as a reference for fold-change in expression using 18S rRNA expression levels as a normalizing endogenous control. Data are represented as fold-change means and standard deviation of three independent experiments performed in duplicate. Asterisks were used to indicate a value of *p* considered statistically significant (* *p* < 0.05).

When the M1 markers’ panel was measured, IL-6 showed statistically significant differences for the three serotypes studied at both times of bacteria stimulation compared with the control condition (*p* = 0.0006, *p* = 0.0060, and *p* = 0.0138 for the serotypes a, b, and c, respectively, at 2 h of infection, and *p* = 0.0005, *p* = 0.0008, and *p* < 0.0001 for the serotypes a, b, and c, respectively, at 24 h). The differences among the serotypes for this target were observed at 2 h of stimulation by just serotypes a (*p* = 0.0050) and b (*p* = 0.0215) over serotype c, without statistically significant differences between them, and at 24 h for the case of serotype c over serotype a (*p* = 0.0275) (Figure 2A).

Regarding the expression levels of HLA/DRA, serotypes a, b, and c were capable of inducing a statistically significant overexpression of this molecule compared to the non-infected condition at both times of infection (*p* = 0.0004, *p* = 0.0110, and *p* = 0.0045 for the serotypes a, b, and c, respectively, at 2 h of infection, and *p* = 0.0042, *p* < 0.0001, and *p* < 0.0001 for the serotypes a, b, and c, respectively, at 24 h). At two hours, we observed higher levels of HLA/DRA expression for serotype a over serotype b (*p* = 0.0303) and for serotype c over serotype b (*p* < 0.0001). On the other hand, at 24 h of stimulation, statistically significant differences in the expression were observed regarding b serotype in comparison with serotype c (*p* = 0.0127) (Figure 2B).

In terms of the expression of CXCL10, data from macrophage cell samples for all serotypes studied showed statistically significant differences in comparison with the non-infected control condition at 2 and 24 h of infection (*p* = 0.0036, *p* = 0.0004, and *p* = 0.0050 for the serotypes a, b, and c, respectively, at 2 h of infection, and *p* < 0.0001 for the three serotypes at 24 h). The statistically significant differences among the serotypes were observed just at 24 h of infection, with a higher expression induced by serotype a over serotype b (*p* = 0.0002) (Figure 2C).

### 3.3. Expression of M2 Markers in Response to Aggregatibacter actinomycetemcomitans Serotypes

**Figure 3:**
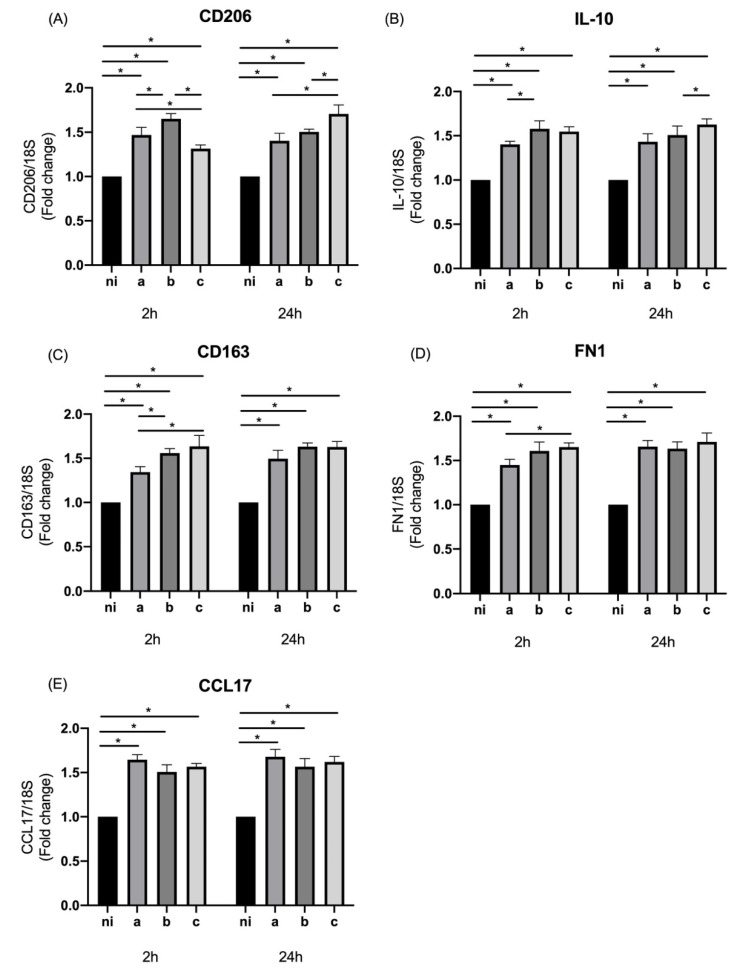
M2 markers’ expression by *Aggregatibacter actinomycetemcomitans*-induced THP-1 human macrophages (ATCC^®^ TIB-202TM). Expression in macrophages infected at an MOI = 102 with strains ATCC^®^ 43717TM (serotype a), ATCC^®^ 43718TM (serotype b), and ATCC^®^ 43719TM (serotype c), 2 and 24 h after infection. For relative expression, mRNA expression in non-infected (ni) macrophages was considered as 1, as a reference for fold-change in expression using 18S rRNA expression levels as a normalizing endogenous control. Data are represented as fold-change means and standard deviation of three independent experiments performed in duplicate. Asterisks were used to indicate a value of *p* considered statistically significant (* *p* < 0.05).

The expression of CD206 in the studied conditions was statistically significantly higher for the three serotypes (*p* < 0.0001) with respect to the control conditions at 2 and 24 h of infection. The statistically significant differences among the serotypes were observed at 2 h of infection between the serotypes a and b (*p* = 0.0109), b and c (*p* < 0.0001), and between the serotypes a and c (*p* = 0.0397), with a higher expression induced by serotype b over the other serotypes. In contrast, at 24 h of infection, statistically significant differences among the serotypes showed a higher expression induced by serotype c over serotypes b (*p* = 0.0001) and c (*p* = 0.0059) (Figure 3A).

The qPCR data reveal that in the case of IL-10, the expression induced by all serotypes studied was statistically significantly higher compared to the control conditions at both times of infection (*p* < 0.0001 for all serotypes). When the differences among serotypes were analyzed, our data showed an expression statistically significantly higher for serotype b over serotype a at 2 h of infection (*p* = 0.0227) and for serotype c over serotype b (*p* = 0.0108) at 24 h of stimulation (Figure 3B).

In terms of the expression of CD163, the three serotypes of A. actinomycetemcomitans studied were capable of inducing an expression statistically significantly higher than the control condition at the two time points of infection (*p* < 0.0001 for all serotypes), and the differences among serotypes for this marker were statistically significant only at 2 h of infection for serotype c over serotype a (*p* = 0.0004) without statistically significant differences between the serotypes at 24 h (Figure 3C).

In the same way, when the levels of expression of FN1 were measured, the three bacteria analyzed in this study induced an expression statistically significantly higher in comparison to the non-infected condition (*p* < 0.0001 for all serotypes) at both times of infection, and differences among the serotypes were only observed at 2 h of infection for serotype c on serotype a (*p* = 0.0103) (Figure 3D), just as happened with CD163.

Finally, we also evaluated the expression of CCL17, by THP-1 cells infected with the three previously mentioned serotypes of *A. actinomycetemcomitans*, observing for both time points studied and all serotypes, the ability to induce a statistically significant overexpression of this marker compared to the uninfected condition (*p* < 0.0001). The analysis of the differences among serotypes did not show a statistically significant difference for any of the evaluated times (Figure 3E).

## 4. Discussion

In periodontitis and other diseases, the innate immune system produces a protective inflammatory response against damage signals, such as pathogens’ presence or tissue destruction, eliminating microorganisms and removing cellular debris to recover cell integrity and maintain homeostasis of the tissues [33].

Macrophage polarization into different subtypes seems to shape macrophage responses and clusters them according to the stimuli, in terms of the diversity of the microenvironment, the amounts of cytokines present in the tissues, and the duration and size of exposure [34]. This polarization has been classified into two major macrophage polarization clusters, classically activated macrophages or M1 and alternatively activated macrophages or M2, each related to both the progression and resolution of inflammation [35].

Classically activated macrophages or M1 constitute the first line of defense with epithelial cells at the level of the periodontal pocket against intracellular pathogens such as *A. actinomycetemcomitans* and promote the Th1 polarization of CD4 cells in in the later stages of the periodontal immune response [36]. This subset of macrophages exhibited a high level of phagocytic activity, and markers that best characterized them were IL-6 and CXCL10; however, the level of expression of these markers is dependent on the nature of the stimulus such as interferon gamma, LPS, or both [37]. However, in our hands, the heterogenic virulence of *A. actinomycetemcomitans* LPS based on the structural differences, under the experimental conditions used, did not show a marked tendency to induce macrophage polarization to an M1 phenotype of one serotype over another when the markers IL-6, CXCL10, and HLA/DRA were measured.

This macrophage phenotype produces proinflammatory cytokines such as IL-1β, IL-6, IL-12, IL-18 and IL-23, TNF-α, and type I IFN; and several chemokines such as CXCL1, CXCL3, CXCL5, CXCL8, CXCL9, CXCL10, CXCL11, CXCL13, and CXCL16; CCL2, CCL3, CCL4, CCL5, CCL8, CCL15, CCL11, CCL19, and CCL20, most of which have been defined as part of the “first wave” of cytokines during the development of periodontal inflammation, acting as the first alarm signal for the assembly of a more complex and specific immune response and for the recruitment of professional immune cells to the site of infection [38].

M2 macrophages are mainly identified based on the expression of CD163, IL-10, CCL17, and other markers [39]. They produce high amounts of IL-8, monocyte chemo-attractant protein-1 (MCP)-1, IP-10, macrophage inflammatory protein (MIP)-1β, and CCL5 or Regulated on Activation, Normal T Cell Expressed and Secreted (RANTES) to recruit neutrophils, monocytes, and T lymphocytes in an antiinflammatory or regulatory response, participating mainly in the remodeling of tissues and the resolution of the inflammatory condition [37,40].

The role of the balance between M1 and M2 macrophages in the onset and development of periodontitis has not been fully studied yet. When the M1/M2 status of macrophage polarization in healthy, gingivitis, and periodontitis patient samples is measured using biopsies, the evidence shows that gingivitis and periodontitis differ from each other by the levels of macrophage infiltrate, but not by changes in macrophage polarization [41] in contrast with some studies that have shown a higher amount of M1 macrophages (higher M1/M2 ratio) in the biopsies of patients with periodontitis compared with healthy or gingivitis individuals [36]. On other hand the presence of M2 macrophages in some study models has been indicative of the role of this cell cluster in the repair process; for instance, the shift in the polarization towards M2 macrophages in the early stage of tissue repair contributed to the enhanced periodontal regeneration after stem cell transplantation in a rat model [42].

When the magnitude of the expression of the panels of markers selected for this study is observed, our data show that the three bacterial serotypes analyzed have the ability to induce a greater expression of markers associated with an M1 phenotype compared to the markers of the M2 phenotype, without marked differences for one serotype over another, adding evidence that the heterogeneity of the virulence of *A. actinomycetemcomitans* cannot be explained, at least in its entirety, by the structural variables of the LPS, and that many other virulence factors are responsible for inducing greater responses against one serotype over another, probably in specialized immune cells capable of discriminating these differences, in contrast to innate immune cells responsible for assembling generic responses.

These results should be analyzed considering the limitations of this study in terms of the use of a single microorganism in an environment of a single cell type, which differs from the real context of periodontitis where the participation of various cell types is conjugated: a biofilm bacterial complex with the presence of cytokines, chemokines, resolvins, metalloproteinases, and other molecules intertwined in a complex network of signals that allow the activation of the mechanisms of the disease and its resolution [38].

Based on the above, we can say that the role of macrophage polarization in periodontal disease is a gap yet to be closed, asking questions such as: How is it that these subpopulations are regulated? What is the role of the subpopulations of M2 macrophages M2a, M2b, and M2c on periodontal diseases? How could the M2b macrophage population, defined with a key role in the response to LPS (a molecule common to most periodontal pathogens), be modulating the inflammatory and tissue destruction mechanisms present in periodontal diseases associated with biofilm [43,44].

Macrophage polarization-modulating drugs might be the future of the immune regulation for the prevention of, treatment, and reduction in patient susceptibility for periodontal diseases [45,46,47,48]. The complete study of the role of macrophages in periodontal disease could be a new area in the study of molecular diagnostics and therapeutic tools, the development of biomarkers, and upgrading the clinical protocols to treat periodontal diseases.

Current studies on the etiopathogenesis of periodontitis demonstrate a constant interaction between microbes and the host inflammatory response as a continuum, or self-sustaining feedback loop, as inflammation provides the anaerobic environment, increased temperature, and vital nutrients, hemin, amino acids, and other growth factors through the gingival crevicular fluid for the growth of bacteria that have been described as “inflammophilics” [49].

In this sense, the clinical relevance and implication of these results allow a greater understanding of the role of inflammatory mechanisms in the chronicity of periodontitis, where, although inflammation is defined as a protective physiological response to infection, alterations in its modulating mechanisms perpetuate the inflammatory picture and favor the development and chronicity of this pathology. It is probable that the polarization of macrophages to a proinflammatory M1 phenotype, induced by keystone pathogenic microorganisms such as *Aggregatibacter actinomycetemcomitans*, is one of the ways by which the inflammatory response is deregulated and becomes sustained over time, preventing the resolution and return of periodontal tissues’ homeostasis and the re-establishment of a truly commensal plaque microbiome that is also in a homeostatic relationship with the host [50].

## 5. Conclusions

The most prevalent serotypes of *A. actinomycetemcomitans* in the THP-1 cell line induce the expression of M1 macrophages markers over the M2 markers in a non-serotype-dependent manner when these cells are stimulated by this pathogen. This indicates a convergence of views with the realization of the critical nexus between periodontal keystone pathogens and inflammation.

These results are in line with the evidence suggesting that the driver of the disease is the inflammation continuum and that only at a late stage do the microbial specificity (pathogenicity) and even the various structures of its virulence factors (as analyzed in this study) begin to play a more prominent role, leaving open the field of research on the mechanisms of the resolution of inflammation and how these could lead to a change in the microbial composition and the restoration of the microbiological balance/homeostasis [51].

**Figure 4:**
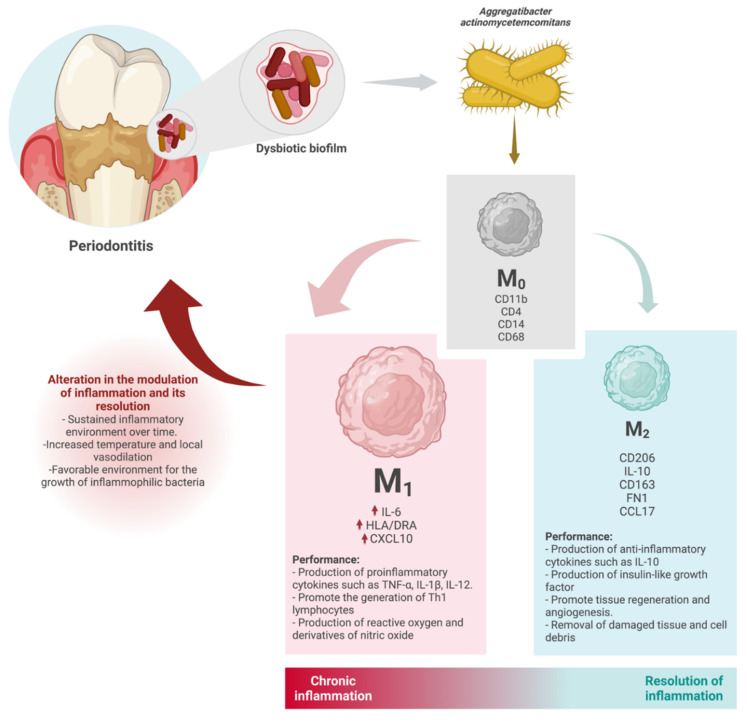
Macrophage polarization towards an M1 phenotype over an M2 phenotype, induced by *Aggregatibacter actinomycetemcomitans*, with increased gene expression of markers such as IL-6, HLA/DRA, and CXCL (characteristic of M1 populations), being one of the possible pathways of dysregulation of inflammation, preventing its resolution and contributing to an environment that favors bacterial dysbiosis in the periodontal microbiome.

The data of this study reinforce the idea that the tendency to polarize macrophages to a proinflammatory M1 phenotype induced by periodontal pathogens such as *Aggregatibacter actinomycetemcomitans* over an M2 phenotype, could be one of the mechanisms of inflammation dysregulation and a driver for the establishment of the disease (Figure 4).

## Figures and Tables

**Table 1:** Sequences of primers used.

Gene	Forward Primer	Reverse Primer
CD11b	cagcctttgaccttatgtcatgg	cctgtgctgtagtcgcact
CD4	cctcctgcttttcattgggctag	tgaggacactggcaggtcttct
CD14	ctggaacaggtgcctaaaggac	gtccagtgtcaggttatccacc
CD68	cgagcatcattctttcaccagct	atgagaggcagcaagatggacc
IL-6	agacagccactcacctcttcag	ttctgccagtgcctctttgctg
HLA-DRA	aaaaggagggagttacactcagg	gctgtgagggacacatcagag
CXCL10	ggtgagaagagatgtctgaatcc	gtccatccttggaagcactgca
CD206	gcaaagtggattacgtgtcttg	ctgttatgtcgctggcaaatg
IL-10	tctccgagatgccttcagcaga	tcagacaaggcttggcaaccca
CD163	ccagtcccaaacactgtcct	atgccagtgagcttcccgttcagc
FN1	acaacaccgaggtgactgagac	ggacacaacgatgcttcctgag
CCL17	ccagggatgccatcgtttttgtaactgtgc	cctcactgtggctcttcttcgtccctggaa
18S	ctcaacacgggaaacctcac	cgctccaccaactaagaacg

Primers were designed using the platform Ensembl Genome and Primer-BLAST (NCBH-NIH).
